# Can Gastric Cancer Patients with High Mandard Score Benefit from Neoadjuvant Chemotherapy?

**DOI:** 10.1155/2022/8178184

**Published:** 2022-03-25

**Authors:** Wen-Zhe Kang, Bing-Zhi Wang, Deng-Feng Li, Zhi-Chao Jiang, Jian-Ping Xiong, Yang Li, Peng Jin, Xin-Xin Shao, Hai-Tao Hu, Yan-Tao Tian

**Affiliations:** ^1^Department of Pancreatic and Gastric Surgery, National Cancer Center/National Clinical Research Center for Cancer/Cancer Hospital, Chinese Academy of Medical Sciences and Peking Union Medical College, Beijing 100021, China; ^2^Department of Pathology, National Cancer Center/National Clinical Research Center for Cancer/Cancer Hospital, Chinese Academy of Medical Sciences and Peking Union Medical College, Beijing 100021, China; ^3^Department of Diagnostic Radiology, National Cancer Center/National Clinical Research Center for Cancer/Cancer Hospital, Chinese Academy of Medical Sciences and Peking Union Medical College, Beijing 100021, China; ^4^Department of Medical Oncology, National Cancer Center/National Clinical Research Center for Cancer/Cancer Hospital, Chinese Academy of Medical Sciences and Peking Union Medical College, Beijing 100021, China

## Abstract

A high Mandard score may indicate the tumor is insensitive to chemotherapy. We analyzed tumor regression and lymph node response under different Mandard scores to assess the impact of Mandard score on prognosis. *Methods*. Mandard scores and ypN stage of postoperative pathological reports were recorded. The results were reviewed by a professional pathologist. The radiologist compared the tumor regression before and after chemotherapy by computed tomography (CT). The survival of all patients was obtained by telephone follow-up. Multivariate Cox regression was used to assess the relationship between overall risk of death and Mandard score, imaging evaluation, and ypN stage. *Results*. In the Mandard score (4-5) group, the median survival time for PR and ypN0 patients was 68.5 and 76.7 months. While in the Mandard score (1-2) group, the median survival time for PD and ypN3a patients was 15.6 and 14.5 months. Imaging evaluation of tumor regression (PR 68.5 months, SD 27.8 months, and PD 10.2 months) and lymph node remission (ypN0 76.7 months, ypN1 61.6 months, ypN2 18.0 months, ypN3a 18.7 months, and ypN3b 18.3 months) showed improved survival. Mandard score, imaging evaluation, and ypN stage are important prognostic factors affecting prognosis. *Conclusion*. A high Mandard score does not mean neoadjuvant chemotherapy is ineffective in gastric cancer. Patients with imaging evaluation of tumor regression and ypN stage reduction may benefit from neoadjuvant chemotherapy.

## 1. Introduction 

Gastric cancer patients with ≥T2 any N+ stage can receive neoadjuvant chemotherapy before surgery [1]. Neoadjuvant chemotherapy increases the chance of therapeutic resection and becomes an important part of the comprehensive treatment of locally advanced gastric cancer [2–5]. Despite some progress, the cure rate (about 40%) remains low [1]. Methods of assessing the efficacy of chemotherapy include the assessment of radiological and histopathological responses.

Mandard tumor regression grade is an important criterion to measure chemotherapy response. Mandard score was obtained by histopathological analysis of the proportion of primary tumors. Mandard 1 describes complete fibrosis (complete pathological response) and Mandard 5 corresponds to no tumor fibrosis (no response to chemotherapy) [6]. Despite high scores, some patients still showed reduced tumor volume and decreased ypN stage. Tumor regression after neoadjuvant chemotherapy as assessed by CT is associated with improved survival [7]. The degree of lymph node fibrosis after chemotherapy does not always correspond to the degree of fibrosis in the primary tumor [8]. Although a high Mandard score (4 and 5) shows the adverse response of tumor tissue to chemotherapy, neoadjuvant chemotherapy may not be ineffective in all cases.

We compared the survival of patients with Mandard score (1-2) and Mandard score (4-5) in different subgroups to study the effect of Mandard tumor regression grade on the prognosis of patients with gastric cancer.

## 2. Methods

### 2.1. Study Design

This is a retrospective cohort study. The study included 393 patients with gastric cancer who were admitted to the National Cancer Center from April 2011 to October 2017. The follow-up ended in April 2020.

### 2.2. Participants

A total of 393 patients were treated at the Department of Pancreatic and Gastric Surgery, National Cancer Center, between April 2011 and October 2017. Criteria for inclusion included pathological examination confirmed primary gastric cancer, the initial clinical stage was cT3-4aN + M0, and received neoadjuvant chemotherapy followed by radical surgical excision + D2 lymph node dissection. The survival of the patients was followed up by telephone until May 2020.

### 2.3. Variables and Measurement

Mandard score and ypN staging were obtained by postoperative pathological reports. The results were reviewed and quality-controlled by professional pathologists. Imaging evaluation was performed by a professional radiologist, and tumor regression was determined by CT comparison before and after neoadjuvant chemotherapy. Mandard scores (4-5) were defined as the nonresponse group, and standard scores (1-2) were defined as the high response group. Radiographic assessment was divided into complete response, partial response, stable, and progressive groups. Imaging evaluation was divided into complete response (CR), partial response (PR), stable disease (SD), and progressive disease (PD) groups. CR was defined as the disappearance of all lesions with no new lesions and a duration of more than 4 weeks. PR was defined as a lesion reduction of more than 30%, lasting more than 4 weeks. SD was defined as a stable lesion with changes between PR and PD. PD is defined as disease progression with an increase of more than 20%. ypN stage was divided into N0, N1, N2, N3a, and N3b groups according to the 8th edition of AJCC guidelines for gastric cancer.

### 2.4. Statistical Methods

The Kaplan–Meier method was used to calculate median survival for different groups. *P* values were calculated by the log-rank test. Multivariable Cox regression analysis further explained the influence of different factors (Mandard score, imaging evaluation, and ypN stage) on prognosis.

Statistical analysis was performed using R software 4.0.5 (R Foundation for Statistical Computing, Vienna, Austria) and the SPSS 22.0 (SPSS Inc., Chicago, IL, USA). Each test was bilateral, and a difference of *P* < 0.05 indicated statistical significance.

## 3. Results

### 3.1. Patient Characteristics

A total of 393 patients were enrolled from 2011 to 2017.  Table 1 provides the baseline characteristics of all patients. 88/393 (22.4%) patients had a Mandard score of 1-2, and 168/393(42.7%) patients had a Mandard score of 4-5. The number and proportion of patients with different imaging evaluation results were the PR group 187 (47.6%), SD group 168 (42.7), and PD group 38 (9.7%). None of the patients were assessed for CR. The number and proportion of patients with different ypN stages were the N0 group 136 (34.6%), N1 group 83 (21.1%), N2 group 68 (17.3%), N3a group 55 (14.0%), and N3b group 51 (13.0%), respectively. Most patients were treated with cisplatin + capecitabine (XP), cisplatin + S-1 (SP), oxaliplatin + capecitabine (XELOX), and oxaliplatin + S-1 (SOX). Some patients were combined with paclitaxel on the basis of a two-drug regimen. Most patients undergo neoadjuvant chemotherapy for 2–6 cycles. 238 (60.6%) patients received adjuvant chemotherapy after surgery.

### 3.2. Survival in Different Groups

**Figure 1:**
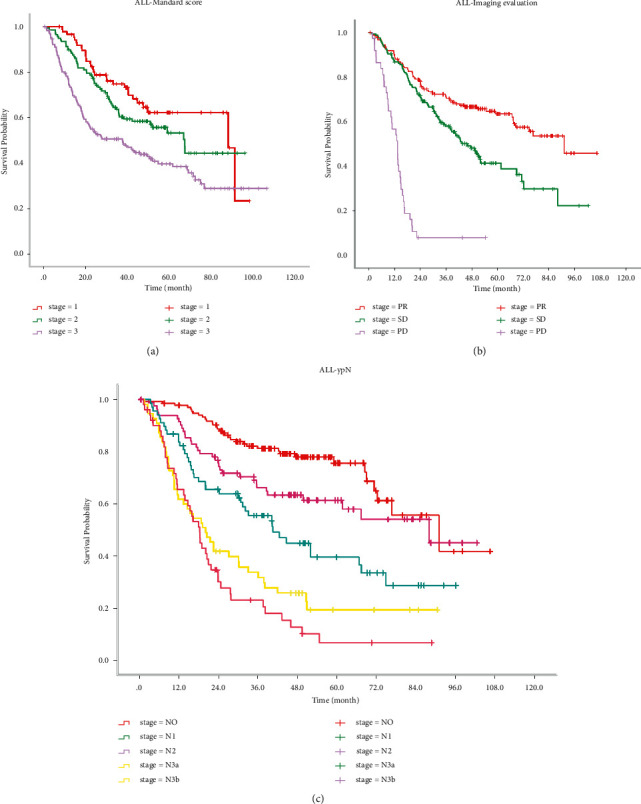
Kaplan–Meier survival analysis for all patients. (a) By the Mandard score group. (b) By the imaging evaluation group. (c) By the ypN group.

**Figure 2:**
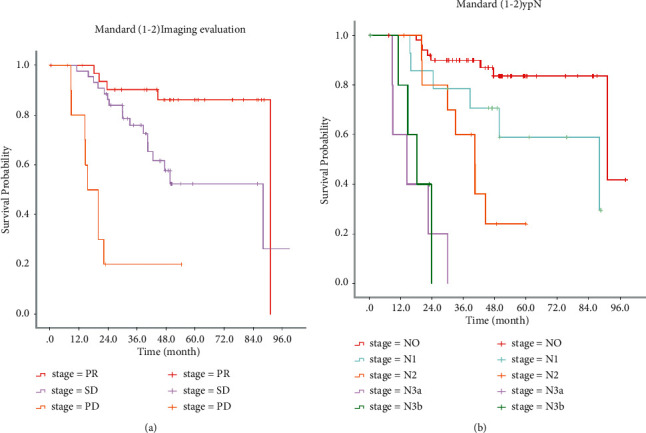
Kaplan–Meier survival analysis for the Mandard score (1-2) group. (a) By the imaging evaluation group. (b) By the ypN group.

**Figure 3:**
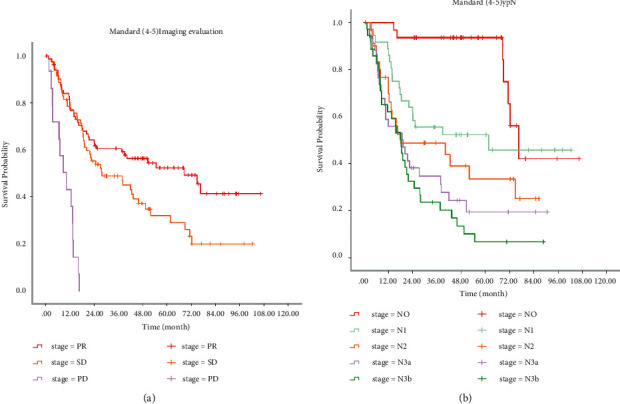
Kaplan–Meier survival analysis for the Mandard score (4-5) group. (a) By the imaging evaluation group. (b) By the ypN group.

Figure 1 shows Kaplan–Meier survival analysis for all patients (a by the Mandard score group, b by the imaging evaluation group, and c by the ypN group).  Figure 2 shows Kaplan–Meier survival analysis for the Mandard score (1-2) group (a by the imaging evaluation group; b by the ypN group).  Figure 3 shows Kaplan–Meier survival analysis for the Mandard score (4-5) group (a by the imaging evaluation group and b by the ypN group).

**Figure 4:**
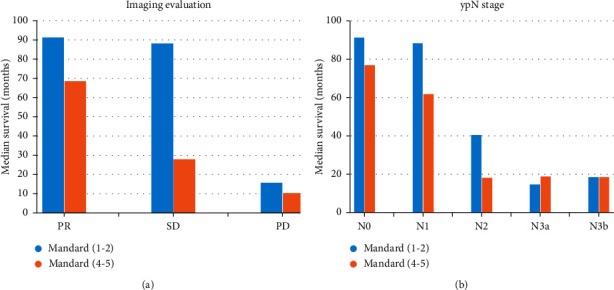
Median survival time for the Mandard score (1-2) group and the Mandard score (4-5) group. (a) By the imaging evaluation group. (b) By the ypN group.

Table 2 provides the median survival time for patients in different subgroups. In all patients, median survival times were 91.1, 46.6, and 13.3 months for PR, SD, and PD groups and 91.1, 88.1, 40.5, 20.2, and 18.3 months for ypN0, ypN1, ypN2, ypN3a, and ypN3b groups. In Mandard (1-2) patients, median survival time was 91.1, 88.1, and 15.6 months for PR, SD, and PD groups and 91.1, 88.1, 40.4, 14.5, and 18.3 months for ypN0, ypN1, ypN2, ypN3a, and ypN3b groups. In Mandard (4-5) patients, median survival times were 68.5, 27.8, and 10.2 months for PR, SD, and PD groups and 76.7, 61.6, 18.0, 18.7, and 18.3 months for ypN0, ypN1, N2, N3a, and N3b groups. The log-rank test showed that there were significant differences among subgroups with different standard scores (*P* < 0.001).  Figure 4 shows the comparison of median survival time for the Mandard score (1-2) group and the Mandard score (4-5) group (a by the imaging evaluation group and b by the ypN group).

Multivariable Cox analysis (Table 3) showed that Mandard score (*P* < 0.001), imaging evaluation of tumor regression (*P* < 0.001), and ypN stage (*P* < 0.001) were independent predictors of prognosis.

## 4. Discussion

Gastric cancer is one of the most common malignant tumors in the world. In recent years, this situation is improving due to the popularity of endoscopy and the development of detection and treatment techniques for *Helicobacter pylori* [9]. Recent studies have shown that both intestinal microbiota and diet affect the occurrence of gastric cancer [10, 11]. Lymph node metastasis is an important factor affecting prognosis. Methods such as the eCura system have been used to evaluate lymph node metastasis [12]. Lymph node metastasis and tumor invasion ≥T2 were considered indications for neoadjuvant chemotherapy [1]. Evaluation of the effect of neoadjuvant chemotherapy is a research hotspot.

Our study found that even with a high Mandard score (4-5), 83 (49.4%) patients had tumor regression (PR) on imaging evaluation and 32 (19.0%) patients had ypN0 stage (from cN + to ypN0). Median survival times for the PR group and ypN0 group were 68.5 and 76.7 months, respectively. Meanwhile, among patients with Mandard score (1-2), the median survival times were 15.6 months in the PD group and 14.5 months in the ypN3a group. Our results suggest that imaging evaluation of tumor regression and remission of lymph node metastasis after neoadjuvant chemotherapy can significantly improve prognosis. For these patients, a high Mandard score does not mean that neoadjuvant chemotherapy is completely ineffective. This finding may be important in clinical decision-making for future treatment plans, such as adjuvant chemotherapy after surgery.

Other studies have similarly concluded that survival is improved in patients who have lymph node responses to chemotherapy, despite poor response to chemotherapy in primary tumors [8, 13–15]. It has been reported that the survival of patients with Mandard score (3–5) in the ypN0 group was similar to that of patients with Mandard score (1-2) [15]. Our study further compared median survival in different ypN stage subgroups of patients with Mandard scores (4-5) and those with Mandard scores (1-2). The survival time of Mandard score (1-2) patients with the ypN2+ stage was much lower than that of Mandard score (4-5) patients with the ypN0 stage. Therefore, some studies suggest that lymph node metastasis and pathological response to chemotherapy are independent predictors of survival after neoadjuvant chemotherapy and surgical resection [16–19].

Radiography plays an important role in evaluating tumor regression after chemotherapy [20–22]. Contrast-enhanced CT after neoadjuvant chemotherapy compared with baseline can effectively predict tumor regression and staging reduction after chemotherapy, which is helpful to propose individualized treatment strategies. Some studies have even suggested that radiomics signature based on computed tomography can predict gastric cancer survival and chemotherapy benefit more accurately than clinicopathological features and TNM staging [23]. Our study also confirmed that imaging evaluation grading can effectively predict the prognosis of patients after neoadjuvant chemotherapy. The median survival time of Mandard score (1-2) patients in the PD group was much lower than that of Mandard score (4-5) patients in the PR group. Among patients with high Mandard scores, patients with imaging evaluation tumor response still benefited from neoadjuvant chemotherapy.

Histopathological measurements of tumor regression provide important information for assessing the efficacy of neoadjuvant chemotherapy. There are several evaluation systems for tumor regression grading. Some assessment systems, such as the Becker system [24], the Chirieac system [25], the Schneider system [26], and the Rizk system [27], are based on the percentage of residual tumor in the lesion. The Mandard score in our study evaluated the efficacy of chemotherapy based on the degree of fibrosis in the primary tumor lesion [28]. There has been some controversy over the accuracy of Mandard scores in evaluating the efficacy of neoadjuvant chemotherapy [29]. Chetty et al. found that the results of Mandard scoring lacked repeatability [30]. One reason for the higher controversy over the Mandard score may be the difficulty of assessing the relative amount of fibrosis [29]. In addition, studies have reported that 40% of patients with lymph node reaction after neoadjuvant chemotherapy had poor response to the primary tumor [8]. PET-CT examination after neoadjuvant chemotherapy showed that the metabolic response of lymph nodes was inconsistent with that of the primary tumor [14]. The probable reason is that lymph node metastasis represents an aggressive cancer clonal subgroup with independent and complex genetic and phenotypic evolution distinct from the primary tumor [31]. These studies suggest that the Mandard grade of tumor degeneration may not be the only criterion for assessing chemotherapy response.

Inconsistencies between image evaluation, ypN staging, and tumor Mandard scores suggest adjuvant and neoadjuvant strategies. Studies have shown that adjuvant chemotherapy has a survival benefit, especially in patients who have already responded to chemotherapy. This benefit may be underestimated if the Mandard grade of tumor degeneration is the sole criterion for assessing chemotherapy response.

There are some limitations to our study. This is a retrospective study, and the results should be interpreted with more caution than prospective studies. Although imaging evaluation is completed by professional radiologists, there is a certain subjective judgment and lack of rigorous quality control. We did not summarize the detailed chemotherapy regimen and cycle for all patients, which may affect the study results. Gastroesophageal junction tumors and distal gastric tumors are different, and we did not discuss them separately. Our data source is a single-center, and a multicenter study with a larger sample is needed to obtain higher-level evidence.

## 5. Conclusion

The evaluation of the neoadjuvant chemotherapy effect is a hot and difficult issue in research. Tumor tissue Mandard score may be inconsistent with ypN staging and imaging evaluation after chemotherapy. Patients with imaging evaluation of tumor regression and ypN stage reduction may benefit from neoadjuvant chemotherapy. Multiple indicators to evaluate the efficacy of neoadjuvant chemotherapy can make patients benefit from subsequent adjuvant chemotherapy.

## Figures and Tables

**Table 1:** Clinicopathological characteristics for all patients.

Clinicopathological characteristics	Mandard (1-2)	Mandard (4-5)	All patients
Number	88	168	393

Sex			
Man	65 (73.9%)	132 (78.6%)	296 (75.3%)
Woman	23 (26.1%)	36 (21.4%)	97 (24.7%)

Number of dissected lymph nodes			
≤15	5 (5.7%)	16 (9.5%)	30 (7.6%)
15–30	41 (45.6%)	71 (42.3%)	170 (43.3%)
>30	42 (47.7%)	81 (48.2%)	193 (49.1%)

Imaging evaluation			
PR	34 (38.6%)	83 (49.4%)	187 (47.6%)
SD	44 (50.0%)	70 (41.7%)	168 (42.7)
PD	10 (11.4%)	15 (8.9%)	38 (9.7%)

ypT stage			
T1	33 (37.5%)	12 (7.2%)	58 (14.8%)
T2	12 (13.6%)	20 (11.9%)	61 (15.5%)
T3	14 (15.9%)	55 (32.7%)	120 (30.5%)
T4	29 (33.0%)	81 (48.2%)	154 (39.2%)

ypN stage			
N0	52 (59.1%)	32 (19.1%)	136 (34.6%)
N1	15 (17.0%)	36 (21.4%)	83 (21.1%)
N2	11 (12.5%)	30 (17.9%)	68 (17.3%)
N3a	5 (5.7%)	34 (20.2%)	55 (14.0%)
N3b	5 (5.7%)	36 (21.4%)	51 (13.0%)

ypTNM			
0-I	41 (46.6%)	20 (11.9%)	82 (20.9%)
II	16 (18.2%)	32 (19.0%)	112 (28.5%)
III	30 (34.1%)	110 (65.5%)	190 (48.3%)
IV	1 (1.1%)	6 (3.6%)	9 (2.3%)

Mandard score			
1	54 (61.4%)	—	54 (13.7%)
2	34 (38.6%)	—	34 (8.7%)
3	—	—	137 (34.9%)
4	—	15 (8.9%)	15 (3.8%)
5	—	153 (91.1%)	153 (38.9%)

Adjuvant chemotherapy			
Yes	48 (54.5%)	90 (53.6%)	238 (60.6%)
No	40 (45.5%)	78 (46.4%)	155 (39.4%)

**Table 2:** Median survival time in patients with different Mandard scores and clinicopathological characteristics.

Mandard score	Category	*N*	Median survival (months)	Log-rank test
All				
	Imaging evaluation			*P* < 0.001
	PR	187	91.1	
	SD	168	46.6	
	PD	38	13.3	
	ypN stage			*P* < 0.001
	N0	136	91.1	
	N1	83	88.1	
	N2	68	40.5	
	N3a	55	20.2	
	N3b	51	18.3	

Mandard (1-2)				
	Imaging evaluation			*P* < 0.001
	PR	34	91.1	
	SD	44	88.1	
	PD	10	15.6	
	ypN stage			*P* < 0.001
	N0	52	91.1	
	N1	15	88.1	
	N2	11	40.4	
	N3a	5	14.5	
	N3b	5	18.3	

Mandard (4-5)				
	Imaging evaluation			*P* < 0.001
	PR	83	68.5	
	SD	70	27.8	
	PD	15	10.2	
	ypN stage			*P* < 0.001
	N0	32	76.7	
	N1	36	61.6	
	N2	30	18.0	
	N3a	34	18.7	
	N3b	36	18.3	

**Table 3:** Multivariable Cox regression analysis of clinicopathologic variables in relation to overall survival.

Clinicopathological features	HR (95% CI)	*P* value
Mandard score		
1-2	Reference	*P* < 0.001
3	2.425 (1.502–3.915)	*P* < 0.001
4-5	3.369 (2.316–5.313)	*P* < 0.001

Imaging evaluation		
PR	Reference	*P* < 0.001
SD	1.491 (1.073–2.072)	*P*=0.017
PD	8.181 (5.133–13.038)	*P* < 0.001

ypN stage		
N0	Reference	*P*=0.503
N1	1.201 (0.703–2.503)	*P* < 0.001
N2	1.922 (1.157–3.194)	*P*=0.012
N3a	2.970 (1.785–4.943)	*P* < 0.001
N3b	3.792 (2.237–6.428)	*P* < 0.001

## Data Availability

The data used to support the findings of this study are included within the supplementary information files.

## Supplementary Materials

Supplementary Materials The data provided in the supplementary files are used to support the findings of this study.

## References

[1] Smyth E. C., Nilsson M., Grabsch H. I., Van Grieken N. C., Lordick F. (2020). Gastric cancer. *Lancet*.

[2] Schuhmacher C., Gretschel S., Lordick F. (2010). Neoadjuvant chemotherapy compared with surgery alone for locally advanced cancer of the stomach and cardia: European organisation for research and treatment of cancer randomized trial 40954. *Journal of Clinical Oncology*.

[3] Kang Y. K., Yook J. H., Park Y. K. (2021). PRODIGY: a phase III study of neoadjuvant docetaxel, oxaliplatin, and S-1 plus surgery and adjuvant S-1 versus surgery and adjuvant S-1 for resectable Advanced gastric cancer. *Journal of Clinical Oncology*.

[4] Allen C. J., Pointer D. T., Blumenthaler A. N. (2021). Chemotherapy versus chemotherapy plus chemoradiation as neoadjuvant therapy for resectable gastric adenocarcinoma: a multi-institutional analysis. *Annals of Surgery*.

[5] Ychou M., Boige V., Pignon J.-P. (2011). Perioperative chemotherapy compared with surgery alone for resectable gastroesophageal adenocarcinoma: an FNCLCC and FFCD multicenter phase III trial. *Journal of Clinical Oncology*.

[6] Knight W. R. C., Baker C. R, Baker C. R. (2021). Does a high Mandard score really define a poor response to chemotherapy in oesophageal adenocarcinoma?. *British Journal of Cancer*.

[7] Li R., Chen T.-W., Hu J. (2013). Tumor volume of resectable adenocarcinoma of the esophagogastric junction at multidetector cT: association with regional lymph node metastasis and N Stage. *Radiology*.

[8] Davies A. R., Myoteri D., Zylstra J. (2018). Lymph node regression and survival following neoadjuvant chemotherapy in oesophageal adenocarcinoma. *British Journal of Surgery*.

[9] Ioannis A. C., Donato D., Skender T., Lucrezia B. (2021). 40 Years of *Helicobacter pylori*: a revolution in biomedical thought. *Gastroenterology Insights*.

[10] Odun-Ayo F., Reddy L. (2022). Gastrointestinal microbiota dysbiosis associated with SARS-CoV-2 infection in colorectal cancer: the implication of probiotics. *Gastroenterology Insights*.

[11] Forma A., Chilimoniuk Z., Januszewski J., Sitarz R. (2021). The potential application of allium extracts in the treatment of gastrointestinal cancers. *Gastroenterology Insights*.

[12] Kazuhiro N, Masahide E, Takaya S, Tomonori Y, Yoshikazu H, Tomohiro I (2022). The modified eCura system for identifying high-risk lymph node metastasis in patients with early gastric cancer resected by endoscopic submucosal dissection. *Gastroenterology Insights*.

[13] Noble F., Lloyd M. A., Turkington R. (2017). Multicentre cohort study to define and validate pathological assessment of response to neoadjuvant therapy in oesophagogastric adenocarcinoma. *British Journal of Surgery*.

[14] Findlay J. M., Bradley K. M., Wang L. M. (2017). Predicting pathologic response of esophageal cancer to neoadjuvant chemotherapy: the implications of metabolic nodal response for personalized therapy. *Journal of Nuclear Medicine*.

[15] Bausys A., Senina V., Luksta M. (2021). Histologic lymph nodes regression after preoperative chemotherapy as prognostic factor in non-metastatic advanced gastric adenocarcinoma. *Journal of Cancer*.

[16] Smyth E. C., Fassan M., Cunningham D. (2016). Effect of pathologic tumor response and nodal status on survival in the medical research council adjuvant gastric infusional chemotherapy trial. *Journal of Clinical Oncology*.

[17] Dhadda A. S., Bessell E. M., Scholefield J., Dickinson P., Zaitoun A. M. (2014). Mandard tumour regression grade, perineural invasion, circumferential resection margin and post-chemoradiation nodal status strongly predict outcome in locally advanced rectal cancer treated with preoperative chemoradiotherapy. *Clinical Oncology*.

[18] Dhadda A. S., Dickinson P., Zaitoun A. M., Gandhi N., Bessell E. M. (2011). Prognostic importance of Mandard tumour regression grade following pre-operative chemo/radiotherapy for locally advanced rectal cancer. *European Journal of Cancer*.

[19] Sada Y. H., Smaglo B. G., Tan J. C., Tran Cao H. S., Musher B. L., Massarweh N. N. (2019). Prognostic value of nodal response after preoperative treatment of gastric adenocarcinoma. *Journal of the National Comprehensive Cancer Network*.

[20] Xu Q., Sun Z., Li X. (2021). Advanced gastric cancer: CT radiomics prediction and early detection of downstaging with neoadjuvant chemotherapy. *European Radiology*.

[21] Li Z.-Y., Wang X.-D., Li M. (2020). Multi-modal radiomics model to predict treatment response to neoadjuvant chemotherapy for locally advanced rectal cancer. *World Journal of Gastroenterology*.

[22] Sun K.-Y., Hu H.-T., Chen S.-L. (2020). CT-based radiomics scores predict response to neoadjuvant chemotherapy and survival in patients with gastric cancer. *BMC Cancer*.

[23] Jiang Y., Chen C., Xie J. (2018). Radiomics signature of computed tomography imaging for prediction of survival and chemotherapeutic benefits in gastric cancer. *E-Bio Medicine*.

[24] Becker K., Mueller J. D., Schulmacher C. (2003). Histomorphology and grading of regression in gastric carcinoma treated with neoadjuvant chemotherapy. *Cancer*.

[25] Chirieac L. R., Swisher S. G., Ajani J. A. (2005). Posttherapy pathologic stage predicts survival in patients with esophageal carcinoma receiving preoperative chemoradiation. *Cancer*.

[26] Schneider P. M., Baldus S. E., Metzger R. (2005). Histomorphologic tumor regression and lymph node metastases determine prognosis following neoadjuvant radiochemotherapy for esophageal cancer. *Annals of Surgery*.

[27] Rizk N. P., Venkatraman E., Bains M. S. (2007). American Joint Committee on Cancer staging system does not accurately predict survival in patients receiving multimodality therapy for esophageal adenocarcinoma. *Journal of Clinical Oncology*.

[28] Mandard A. M., Dalibard F., Mandard J. C. (1994). Pathologic assessment of tumor regression after preoperative chemoradiotherapy of esophageal carcinoma. *Clinicopathologic Correlations Cancer*.

[29] Karamitopoulou E., Thies S., Zlobec I. (2014). Assessment of tumor regression of esophageal adenocarcinomas after neoadjuvant chemotherapy. *The American Journal of Surgical Pathology*.

[30] Chetty R., Gill P., Govender D. (2012). International study group on rectal cancer regression grading: interobserver variability with commonly used regression grading systems. *Human Pathology*.

[31] Jones S., Chen W.-D., Parmigiani G. (2008). Comparative lesion sequencing provides insights into tumor evolution. *Proceedings of the National Academy of Sciences*.

