# Supplementary material for: Can Gastric Cancer Patients with High Mandard Score Benefit from Neoadjuvant Chemotherapy?
Source: Can J Gastroenterol Hepatol. 2022 Mar 25;2022:8178184. doi: 10.1155/2022/8178184 (PMC8975703; doi:10.1155/2022/8178184)
Supplement: Supplementary Materials — The data provided in the supplementary files are used to support the findings of this study. [file 8178184.f1.pdf]

Mandard score	Mandard 分组	T	N	M	survival time	Stage	分期	清扫淋巴结	阳性淋巴结
1	1	Tis	N1	M0	0.5	IB	1	89	2
5	3	T3	N0	M0	106.5	IIA	2	26	0
2	1	T4a	N1	M0	15.63	IIIA	3	10	2
5	3	T1a	N3b	M0	88.73	IIIB	3	28	16
5	3	T2	N2	M0	84.8	IIB	2	37	3
5	3	T2	N0	M0	65.6	IB	1	46	0
3	2	T3	N0	M0	52.07	IIA	2	20	0
3	2	T3	N2	M0	51.9	IIIA	3	30	5
3	2	T3	N0	M0	52.03	IIA	2	32	0
3	2	T2	N0	M0	51.97	IB	1	51	0
4	3	T3	N3a	M0	51.9	IIIB	3	45	7
3	2	T3	N1	M0	51.2	IIB	2	17	1
5	3	T2	N2	M0	42.53	IIB	2	32	4
5	3	T4b	N1	M0	51.47	IIIB	3	33	1
3	2	T2	N2	M0	51.4	IIB	2	20	3
1	1	Tis	N1	M0	49.73	IB	1	44	1
4	3	T1b	N3b	M0	37.57	IIIB	3	63	17
1	1	Tis	N1	M0	45.73	IB	1	21	1
1	1	T1b	N0	M0	39.5	IA	1	23	0
1	1	T1a	N3b	M0	22.97	IIIB	3	49	18
3	2	T3	N2	M0	92.43	IIIA	3	41	4
5	3	T3	N2	M0	74	IIIA	3	21	4
2	1	T4b	N0	M0	42.6	IIIA	3	29	0
5	3	T4a	N1	M0	81.33	IIIA	3	36	1
5	3	T4a	N2	M0	13.77	IIIA	3	40	6
3	2	T3	N1	M0	35.7	IIB	2	20	2
3	2	T3	N1	M0	43.5	IIB	2	17	1
3	2	T4a	N0	M0	35.9	IIB	2	27	0
3	2	T3	N1	M0	5.77	IIB	2	58	2
5	3	T2	N0	M0	68.1	IB	1	32	0
5	3	T4b	N3a	M0	4.17	IIIC	3	38	15
3	2	T3	N0	M0	15.63	IIA	2	21	0
1	1	T2	N0	M0	59.23	IB	1	45	0
5	3	T4b	N1	M0	24.1	IIIB	3	20	2
5	3	T4a	N3b	M0	27.57	IIIC	3	58	19
3	2	T4a	N3b	M0	16.33	IIIC	3	42	31
2	1	T4a	N3b	M0	11.13	IIIC	3	45	38
2	1	T4a	N3b	M0	14.77	IIIC	3	37	31
5	3	T4a	N2	M0	6.47	IIIA	3	23	6
5	3	T4a	N3b	M0	7.8	IIIC	3	31	27
3	2	T4a	N1	M0	34.8	IIIA	3	47	2
1	1	T4a	N3a	M0	9	IIIB	3	36	15
5	3	T4a	N3b	M0	18.33	IIIC	3	55	35
5	3	T4a	N1	M0	17.7	IIIA	3	26	1
3	2	T3	N3a	M0	11.5	IIIB	3	39	7
5	3	T3	N3b	M0	13.5	IIIC	3	26	21
5	3	T3	N3b	M0	46	IIIC	3	56	38
3	2	T3	N3a	M0	8	IIIB	3	21	10
5	3	T4b	N0	M0	68.5	IIIA	3	17	0

5	3	T4a	N1	M0	24	IIIA	3	12	2
5	3	T3	N2	M0	18	IIIA	3	22	5
5	3	T3	N3b	M0	8.6	IIIC	3	30	20
3	2	T3	N2	M0	96.1	IIIA	3	38	6
5	3	T3	N3a	M0	10.17	IIIB	3	25	12
5	3	T4a	N3b	M0	21.8	IIIC	3	38	34
1	1	T4b	N0	M0	47.5	IIIA	3	37	0
5	3	T4b	N0	M0	16.27	IIIA	3	22	0
5	3	T3	N2	M0	74.87	IIIA	3	41	5
5	3	T3	N3b	M0	54.67	IIIC	3	21	17
5	3	T2	N3a	M0	21.5	IIIA	3	20	14
5	3	T4a	N1	M0	12	IIIA	3	18	1
3	2	T4a	N2	M0	8.43	IIIA	3	38	5
5	3	T4a	N2	M0	7.97	IIIA	3	13	6
5	3	T3	N3a	M1	6.97	IV	4	26	7
5	3	T3	N3a	M0	8.8	IIIB	3	23	12
5	3	T3	N3a	M0	90.53	IIIB	3	46	7
3	2	T3	N3a	M0	2.6	IIIB	3	11	11
2	1	T4a	N1	M0	88.1	IIIA	3	35	1
5	3	T3	N3a	M0	6	IIIB	3	14	7
3	2	T4a	N2	M0	5.53	IIIA	3	17	4
1	1	T3	N3a	M0	14.5	IIIB	3	17	7
5	3	T3	N2	M0	86.5	IIIA	3	24	6
3	2	T4a	N1	M0	30.47	IIIA	3	36	2
1	1	T4b	N0	M0	85.73	IIIA	3	25	0
3	2	T2	N3a	M0	16.2	IIIA	3	45	10
3	2	T4b	N2	M0	85.5	IIIB	3	24	3
5	3	T4a	N2	M0	52	IIIA	3	58	3
5	3	T2	N3a	M0	84.8	IIIA	3	57	7
3	2	T4a	N2	M0	14.9	IIIA	3	16	4
3	2	T4b	N1	M0	82.83	IIIB	3	11	2
1	1	T4a	N2	M0	20	IIIA	3	13	5
5	3	T4a	N3a	M0	1.47	IIIB	3	18	12
3	2	T2	N3a	M0	82.13	IIIA	3	18	9
5	3	T4a	N1	M0	5.37	IIIA	3	16	2
5	3	T4a	N3b	M0	4.2	IIIC	3	22	21
5	3	T4a	N3b	M0	7.5	IIIC	3	44	30
5	3	T3	N3b	M0	49.43	IIIC	3	36	24
2	1	T4b	N0	M0	23.67	IIIA	3	20	0
5	3	T4b	N0	M0	25.63	IIIA	3	45	0
5	3	T3	N2	M0	77.13	IIIA	3	18	3
5	3	T4a	N3b	M0	18.5	IIIC	3	32	16
5	3	T3	N3a	M0	38.1	IIIB	3	32	9
5	3	T4a	N3b	M0	43.33	IIIC	3	36	27
5	3	T4a	N3a	M1	2.63	IV	4	13	11
5	3	T4a	N1	M0	50.97	IIIA	3	14	1
5	3	T4a	N3a	M0	41.93	IIIB	3	23	7
5	3	T4a	N1	M0	48	IIIA	3	15	2
3	2	T4b	N3a	M0	50.93	IIIC	3	36	8
5	3	T4a	N1	M0	18.37	IIIA	3	26	1
5	3	T4a	N3b	M0	16	IIIC	3	29	16
5	3	T3	N2	M0	16.57	IIIA	3	19	6
5	3	T3	N3a	M0	10.6	IIIB	3	30	7
5	3	T3	N2	M0	72.3	IIIA	3	37	4

3	2	T3	N3b	M0	38.27	IIIC	3	48	16
5	3	T3	N3a	M0	71.33	IIIB	3	24	14
5	3	T4a	N3b	M0	1.33	IIIC	3	41	34
5	3	T4a	N3b	M0	70.57	IIIC	3	52	22
5	3	T4b	N3b	M0	4	IIIC	3	36	26
3	2	T3	N2	M0	70.1	IIIA	3	57	6
5	3	T4a	N3a	M0	6	IIIB	3	25	11
5	3	T3	N2	M0	12.3	IIIA	3	38	4
5	3	T4a	N1	M0	17.3	IIIA	3	17	1
5	3	T4a	N3b	M0	11.43	IIIC	3	42	18
5	3	T4b	N2	M0	3.3	IIIB	3	18	4
3	2	T4a	N2	M0	67.4	IIIA	3	24	4
3	2	T4a	N1	M0	65.03	IIIA	3	33	2
3	2	T3	N2	M0	14.5	IIIA	3	54	3
5	3	T3	N3a	M0	10.6	IIIB	3	34	15
5	3	T3	N2	M0	15.77	IIIA	3	21	4
5	3	T4a	N3b	M0	2.5	IIIC	3	90	69
5	3	T4b	N3a	M0	11.83	IIIC	3	27	9
5	3	T3	N2	M0	35.5	IIIA	3	36	3
5	3	T4b	N3b	M0	3.33	IIIC	3	41	39
2	1	T4a	N2	M0	44.63	IIIA	3	39	3
1	1	T4a	N3b	M1	18.3	IV	4	43	17
5	3	T4a	N3b	M1	1.6	IV	4	28	28
5	3	T4a	N3b	M0	19	IIIC	3	22	18
1	1	T4b	N2	M0	59.97	IIIB	3	43	5
5	3	T4b	N3b	M0	7.87	IIIC	3	53	23
3	2	T4a	N3b	M0	23.9	IIIC	3	52	22
5	3	T3	N3a	M0	19.2	IIIB	3	14	7
5	3	T4a	N2	M0	4.17	IIIA	3	34	3
3	2	T3	N3a	M0	58.77	IIIB	3	58	7
5	3	T4a	N3b	M0	21	IIIC	3	70	43
1	1	T3	N3b	M0	23.87	IIIC	3	34	26
3	2	T3	N2	M0	15.57	IIIA	3	37	4
5	3	T4a	N2	M0	7.47	IIIA	3	25	6
2	1	T4a	N3a	M0	22.5	IIIB	3	30	12
2	1	T4b	N2	M0	13.3	IIIB	3	26	5
5	3	T4a	N2	M0	11.9	IIIA	3	47	5
3	2	T4a	N3b	M0	6.57	IIIC	3	53	44
4	3	T4b	N3b	M0	6.3	IIIC	3	39	30
5	3	T4a	N2	M0	40.17	IIIA	3	47	4
3	2	T4b	N3b	M0	20.47	IIIC	3	28	16
2	1	T4b	N0	M0	54.03	IIIA	3	31	0
3	2	T4a	N1	M0	54.07	IIIA	3	36	2
5	3	T4a	N3a	M0	18.73	IIIB	3	63	13
5	3	T3	N2	M0	3.87	IIIA	3	21	5
5	3	T4a	N2	M0	50.27	IIIA	3	32	3
1	1	T4a	N1	M0	50	IIIA	3	28	1
3	2	T3	N2	M0	49.83	IIIA	3	49	4
5	3	T4a	N3a	M0	50.6	IIIB	3	19	15
3	2	T4a	N3a	M0	49.43	IIIB	3	45	9
5	3	T4a	N1	M0	25.17	IIIA	3	24	2
2	1	T4a	N1	M0	38.63	IIIA	3	25	2
3	2	T4b	N2	M0	32.2	IIIB	3	39	4
5	3	T3	N3b	M0	24.7	IIIC	3	35	19

5	3	T4a	N3a	M0	7.47	IIIB	3	38	8
3	2	T2	N3a	M0	48.3	IIIA	3	28	11
3	2	T3	N3a	M0	48.5	IIIB	3	17	10
5	3	T3	N3b	M0	17.93	IIIC	3	34	29
5	3	T4a	N3a	M0	47.57	IIIB	3	24	8
5	3	T4b	N2	M0	11.9	IIIB	3	40	6
5	3	T3	N3a	M0	37.8	IIIB	3	74	13
3	2	T4a	N1	M0	22.97	IIIA	3	44	1
3	2	T4a	N3b	M0	11.53	IIIC	3	31	16
5	3	T4a	N3a	M0	8.37	IIIB	3	33	13
5	3	T4a	N3a	M0	46.1	IIIB	3	65	10
5	3	T3	N3a	M0	20.17	IIIB	3	20	11
5	3	T4a	N1	M0	45.5	IIIA	3	31	2
4	3	T3	N3a	M0	15.5	IIIB	3	38	8
4	3	T4a	N3a	M0	27.17	IIIB	3	28	7
5	3	T4a	N1	M0	45.37	IIIA	3	28	1
3	2	T4a	N3b	M1	10.57	IV	4	35	17
3	2	T4a	N3b	M0	49.5	IIIC	3	50	21
2	1	T4a	N2	M0	20.13	IIIA	3	32	5
2	1	T3	N2	M0	40.5	IIIA	3	26	5
3	2	T3	N3a	M0	36.17	IIIB	3	21	10
3	2	T3	N3b	M0	13.83	IIIC	3	43	28
3	2	T3	N3a	M0	10.53	IIIB	3	16	13
2	1	T4a	N3a	M0	8.83	IIIB	3	33	11
3	2	T4b	N0	M0	27.67	IIIA	3	16	0
3	2	T4a	N3a	M0	30.3	IIIB	3	14	12
3	2	T4a	N3a	M0	20.4	IIIB	3	44	7
1	1	T3	N2	M0	40.37	IIIA	3	22	3
3	2	T3	N2	M0	40.23	IIIA	3	22	3
5	3	T4a	N3b	M0	15.4	IIIC	3	67	37
5	3	T4a	N1	M0	2.8	IIIA	3	72	2
5	3	T4a	N3a	M1	6.43	IV	4	21	8
2	1	T4a	N2	M0	39.07	IIIA	3	27	5
1	1	T4a	N3a	M0	30.1	IIIB	3	59	15
3	2	T3	N2	M0	37.87	IIIA	3	36	6
5	3	T3	N3a	M0	22.53	IIIB	3	15	13
3	2	T4a	N3a	M1	33.13	IV	4	54	13
3	2	T4a	N2	M0	37.5	IIIA	3	43	3
4	3	T4a	N3b	M0	27.83	IIIC	3	33	28
3	2	T4a	N2	M0	37.2	IIIA	3	41	3
5	3	T4a	N1	M0	34.9	IIIA	3	40	1
5	3	T4a	N1	M0	34.7	IIIA	3	26	1
2	1	T4a	N1	M0	15.9	IIIA	3	32	2
3	2	T4a	N2	M0	34.87	IIIA	3	13	5
5	3	T4a	N3b	M0	33.77	IIIC	3	37	21
1	1	T4b	N0	M0	33.37	IIIA	3	26	0
5	3	T4a	N3b	M0	8.5	IIIC	3	49	26
4	3	T4a	N3b	M1	3.17	IV	4	47	29
3	2	T3	N3a	M0	13.33	IIIB	3	68	11
5	3	T3	N2	M0	10.3	IIIA	3	18	5
1	1	T4b	N0	M0	30.5	IIIA	3	44	0
5	3	T4a	N3b	M0	7.1	IIIC	3	39	20
1	1	T4b	N2	M0	30	IIIB	3	45	4
5	3	T4b	N2	M1	30.3	IV	4	32	6

5	3	T3	N2	M0	29.9	IIIA	3	21	3
5	3	T4a	N2	M0	29.4	IIIA	3	49	6
5	3	T4a	N3b	M0	20.2	IIIC	3	36	24
5	3	T4a	N3a	M0	5.23	IIIB	3	11	9
5	3	T3	N3a	M0	24.47	IIIB	3	27	7
3	2	T3	N2	M0	23.8	IIIA	3	34	3
3	2	T4a	N3b	M0	23.57	IIIC	3	21	20
5	3	T3	N3a	M0	23.3	IIIB	3	46	9
5	3	T3	N1	M0	102.5	IIB	2	23	2
3	2	T4a	N0	M0	23.5	IIB	2	21	0
5	3	T2	N1	M0	98.1	IIA	2	21	1
5	3	T3	N1	M0	11.6	IIB	2	32	1
4	3	T3	N1	M0	94.4	IIB	2	12	1
2	1	T3	N0	M0	91.1	IIA	2	28	0
3	2	T2	N2	M0	16.4	IIB	2	15	5
3	2	T3	N1	M0	67.33	IIB	2	12	2
5	3	T3	N1	M0	87.9	IIB	2	10	1
3	2	T3	N1	M0	87.93	IIB	2	32	2
5	3	T3	N1	M0	13.8	IIB	2	26	2
3	2	T2	N1	M0	83.13	IIA	2	21	1
3	2	T3	N1	M0	80.63	IIB	2	13	1
3	2	T1a	N2	M0	66.8	IIA	2	38	3
3	2	T4a	N0	M0	1.43	IIB	2	58	0
5	3	T4a	N0	M0	76.67	IIB	2	12	0
3	2	T3	N0	M0	76.6	IIA	2	20	0
2	1	T3	N1	M0	75.53	IIB	2	19	1
5	3	T3	N0	M0	75.4	IIA	2	24	0
3	2	T4a	N0	M0	42.27	IIB	2	36	0
5	3	T3	N1	M0	12.5	IIB	2	17	1
5	3	T3	N1	M0	4.4	IIB	2	60	1
3	2	T2	N1	M0	23.8	IIA	2	28	1
5	3	T3	N0	M0	72.3	IIA	2	41	0
5	3	T3	N1	M0	61.63	IIB	2	13	1
5	3	T3	N0	M0	71.33	IIA	2	25	0
3	2	T4a	N0	M0	29.4	IIB	2	37	0
3	2	T3	N0	M0	25.2	IIA	2	27	0
3	2	T3	N0	M0	69.2	IIA	2	40	0
3	2	T1b	N2	M0	69.17	IIA	2	19	3
4	3	T3	N1	M0	38.93	IIB	2	20	1
5	3	T4a	N0	M0	68.97	IIB	2	50	0
3	2	T4a	N0	M0	32.63	IIB	2	32	0
5	3	T1b	N2	M0	13.37	IIA	2	39	6
3	2	T3	N0	M0	19.13	IIA	2	16	0
3	2	T2	N2	M0	24.2	IIB	2	40	6
1	1	T3	N1	M0	61.27	IIB	2	37	1
3	2	T4a	N0	M0	60.77	IIB	2	47	0
5	3	T2	N1	M0	60.3	IIA	2	10	1
2	1	T4a	N0	M0	18.03	IIB	2	20	0
3	2	T4a	N0	M0	59.7	IIB	2	33	0
1	1	T3	N0	M0	59.67	IIA	2	39	0
5	3	T3	N1	M0	63.47	IIB	2	33	2
3	2	T3	N0	M0	58.23	IIA	2	28	0
3	2	T4a	N0	M0	57.4	IIB	2	31	0
3	2	T4a	N0	M0	57.4	IIB	2	45	0

3	2	T3	N0	M0	57.97	IIA	2	41	0
3	2	T4a	N0	M0	24.27	IIB	2	17	0
3	2	T2	N1	M0	54.2	IIA	2	27	1
5	3	T3	N1	M0	13.93	IIB	2	45	2
3	2	T2	N1	M0	53.83	IIA	2	26	1
3	2	T4a	N0	M0	54.6	IIB	2	24	0
5	3	T3	N0	M0	53.03	IIA	2	11	0
3	2	T1b	N2	M0	54	IIA	2	29	5
3	2	T4a	N0	M0	15.93	IIB	2	53	0
3	2	T4a	N0	M0	51.07	IIB	2	61	0
4	3	T3	N1	M0	51.1	IIB	2	46	2
1	1	T3	N0	M0	50	IIA	2	20	0
3	2	T3	N1	M0	35.67	IIB	2	57	2
3	2	T2	N1	M0	49.1	IIA	2	23	2
1	1	T2	N1	M0	49	IIA	2	45	2
2	1	T2	N2	M0	48.77	IIB	2	30	3
5	3	T2	N2	M0	6.1	IIB	2	36	5
3	2	T3	N0	M0	31.6	IIA	2	17	0
1	1	T3	N0	M0	49.6	IIA	2	15	0
4	3	T4a	N0	M0	47.77	IIB	2	29	0
3	2	T3	N0	M0	7.03	IIA	2	16	0
3	2	T3	N1	M0	47.37	IIB	2	25	1
2	1	T3	N1	M0	47.03	IIB	2	42	2
5	3	T2	N1	M0	46.9	IIA	2	33	2
3	2	T2	N1	M0	46.23	IIA	2	24	1
3	2	T3	N1	M0	45.87	IIB	2	33	1
3	2	T3	N0	M0	45.9	IIA	2	36	0
3	2	T3	N0	M0	44.7	IIA	2	24	0
2	1	T3	N0	M0	20.33	IIA	2	23	0
3	2	T3	N0	M0	44.73	IIA	2	37	0
2	1	T4a	N0	M0	44.3	IIB	2	36	0
3	2	T3	N0	M0	27	IIA	2	17	0
4	3	T4a	N0	M0	43.83	IIB	2	39	0
3	2	T2	N1	M0	41.13	IIA	2	56	1
3	2	T3	N0	M0	41.03	IIA	2	32	0
3	2	T2	N1	M0	12.97	IIA	2	28	2
5	3	T2	N1	M0	22.17	IIA	2	33	1
5	3	T4a	N0	M0	39.37	IIB	2	25	0
2	1	T4a	N0	M0	20	IIB	2	44	0
3	2	T1b	N2	M0	31.37	IIA	2	35	5
3	2	T2	N1	M0	5.57	IIA	2	14	1
5	3	T3	N0	M0	38.27	IIA	2	31	0
3	2	T3	N0	M0	37.73	IIA	2	29	0
2	1	T3	N0	M0	34.57	IIA	2	22	0
3	2	T3	N0	M0	34.33	IIA	2	39	0
2	1	T2	N2	M0	33.13	IIB	2	32	3
3	2	T2	N2	M0	30.8	IIB	2	33	3
3	2	T3	N1	M0	30.8	IIB	2	19	1
3	2	T3	N0	M0	30.6	IIA	2	18	0
1	1	T3	N1	M0	29.8	IIB	2	27	1
3	2	T4a	N0	M0	26.03	IIB	2	53	0
5	3	T4a	N0	M0	25.9	IIB	2	38	0
3	2	T3	N0	M0	22.23	IIA	2	51	0
3	2	T2	N1	M0	25.37	IIA	2	51	2

5	3	T2	N0	M0	14.67	IB	1	19	0
4	3	T1b	N1	M0	13.3	IB	1	25	2
1	1	T2	N0	M0	98.13	IB	1	19	0
5	3	T2	N0	M0	95.43	IB	1	43	0
5	3	T1b	N1	M0	94.17	IB	1	31	1
1	1	T2	N0	M0	50.9	IB	1	28	0
1	1	T1a	N1	M0	88.57	IB	1	21	1
2	1	T1a	N0	M0	87.33	IA	1	30	0
2	1	T1b	N0	M0	85.83	IA	1	32	0
1	1	T1b	N0	M0	85.5	IA	1	26	0
3	2	T1b	N1	M0	85.17	IB	1	22	1
1	1	T1a	N0	M0	79.93	IA	1	26	0
5	3	T1a	N0	M0	79.87	IA	1	35	0
5	3	T2	N0	M0	72.4	IB	1	47	0
3	2	T1b	N0	M0	72.73	IA	1	39	0
3	2	T2	N0	M0	71.87	IB	1	24	0
1	1	T1b	N0	M0	34.5	IA	1	26	0
5	3	T1b	N0	M0	67.83	IA	1	17	0
1	1	T1a	N0	M0	60.3	IA	1	20	0
3	2	T1b	N1	M0	59.5	IB	1	28	1
2	1	T2	N0	M0	59.03	IB	1	58	0
5	3	T1b	N0	M0	58.5	IA	1	16	0
3	2	T2	N0	M0	59.03	IB	1	16	0
3	2	T2	N0	M0	57.53	IB	1	43	0
5	3	T2	N0	M0	56.7	IB	1	16	0
3	2	T1b	N1	M0	56.47	IB	1	42	1
4	3	T2	N0	M0	56.2	IB	1	16	0
1	1	T1a	N0	M0	53.07	IA	1	39	0
3	2	T2	N0	M0	52.53	IB	1	20	0
1	1	T1b	N0	M0	50.53	IA	1	35	0
2	1	T2	N0	M0	50.53	IB	1	64	0
2	1	T1b	N0	M0	50.27	IA	1	21	0
3	2	T2	N0	M0	49.63	IB	1	18	0
5	3	T1a	N0	M0	49.17	IA	1	27	0
1	1	T1a	N0	M0	49.67	IA	1	16	0
5	3	T2	N0	M0	48.23	IB	1	18	0
1	1	T2	N0	M0	47.8	IB	1	37	0
2	1	T1a	N0	M0	47.13	IA	1	18	0
3	2	T1a	N1	M0	47.07	IB	1	76	2
5	3	T2	N0	M0	45.73	IB	1	101	0
3	2	T2	N0	M0	44.8	IB	1	28	0
3	2	T2	N0	M0	43.57	IB	1	46	0
4	3	T2	N0	M0	42.87	IB	1	24	0
1	1	T2	N0	M0	39.97	IB	1	37	0
1	1	T1b	N0	M0	39.8	IA	1	16	0
3	2	T1b	N0	M0	39.47	IA	1	38	0
5	3	T1b	N0	M0	0.5	IA	1	34	0
3	2	T2	N0	M0	12	IB	1	25	0
3	2	T1b	N0	M0	33.9	IA	1	26	0
3	2	T2	N0	M0	10.73	IB	1	28	0
2	1	T1b	N0	M0	29.93	IA	1	46	0
1	1	T1b	N0	M0	29.7	IA	1	20	0
3	2	T2	N0	M0	29.43	IB	1	12	0
5	3	T1b	N1	M0	25.67	IB	1	14	2

1	1	T2	N0	M0	25.1	IB	1	36	0
1	1	T1b	N0	M0	25.13	IA	1	34	0
5	3	T2	N0	M0	24.77	IB	1	10	0
1	1	T1a	N1	M0	24.47	IB	1	39	1
1	1	T2	N0	M0	22.33	IB	1	23	0
3	2	T2	N0	M0	23.43	IB	1	21	0
1	1	T1a	N0	M0	23.33	IA	1	24	0
3	2	T1b	N0	M0	23.3	IA	1	18	0
3	2	T1b	N1	M0	22.9	IB	1	36	1
1	1	Tis	N0	M0	37.9	0 stage	1	12	0
1	1	Tis	N0	M0	7.5	0 stage	1	27	0
1	1	Tis	N0	M0	75.1	0 stage	1	25	0
1	1	Tis	N0	M0	64.07	0 stage	1	18	0
1	1	Tis	N0	M0	54.37	0 stage	1	38	0
1	1	Tis	N0	M0	47.5	0 stage	1	47	0
1	1	Tis	N0	M0	45.5	0 stage	1	43	0
5	3	Tis	N0	M0	44.23	0 stage	1	38	0
1	1	Tis	N0	M0	37.33	0 stage	1	23	0
1	1	Tis	N0	M0	32	0 stage	1	11	0
2	1	Tis	N0	M0	26.93	0 stage	1	33	0

生存状态2死亡	阳性淋巴结分组	清扫个数分级≤15=1，≤30=2，＞30=3	无反应组生存状态2死亡	无反应组时间	无反应组清扫淋巴结分组	无反应组阳性淋巴结分组	无反应组影像学评分
1	1	3	1	106.5	2	0	1
1	0	2	1	88.73	2	4	1
2	1	1	1	84.8	3	2	1
1	4	2	1	65.6	3	0	1
1	2	3	1	51.9	3	3	2
1	0	3	2	42.53	3	2	2
1	0	2	1	51.47	3	1	2
2	2	2	2	37.57	3	4	1
1	0	3	1	74	2	2	1
1	0	3	1	81.33	3	1	2
1	3	3	2	13.77	3	2	2
1	1	2	1	68.1	3	0	1
2	2	3	2	4.17	3	3	1
1	1	3	2	24.1	2	1	1
1	2	2	2	27.57	3	4	2
2	1	3	2	6.47	2	2	3
2	4	3	2	7.8	3	4	1
1	1	2	2	18.33	3	4	2
1	0	2	2	17.7	2	1	2
1	4	3	2	13.5	2	4	3
1	2	3	2	46	3	4	2
1	2	2	2	68.5	2	0	1
2	0	2	2	24	1	1	1
1	1	3	2	18	2	2	1
2	2	3	2	8.6	2	4	1
2	1	2	2	10.17	2	3	3
1	1	2	2	21.8	3	4	2
2	0	2	2	16.27	2	0	1
2	1	3	2	74.87	3	2	1
1	0	3	2	54.67	2	4	1
2	3	3	2	21.5	2	3	1
2	0	2	2	12	2	1	1
1	0	3	2	7.97	2	2	2
2	1	2	2	6.97	2	3	3
2	4	3	2	8.8	2	3	2
2	4	3	1	90.53	3	3	1
2	4	3	2	6	1	3	1
2	4	3	1	86.5	2	2	2
2	2	2	2	52	3	2	2
2	4	3	1	84.8	3	3	1
2	1	3	2	1.47	2	3	1
2	3	3	2	5.37	2	1	2
2	4	3	2	4.2	2	4	1
2	1	2	2	7.5	2	4	2
2	3	3	2	49.43	3	4	2
2	4	2	1	25.63	3	0	1
2	4	3	1	77.13	2	2	2
2	3	2	2	18.5	3	4	2
2	0	2	2	38.1	3	3	2

2	1	1	2	43.33	3	4	2
2	2	2	2	2.63	1	3	3
2	4	2	1	50.97	1	1	2
1	2	3	2	41.93	2	3	2
2	3	2	1	48	1	1	2
2	4	3	2	18.37	2	1	1
2	0	3	2	16	2	4	3
2	0	2	2	16.57	2	2	3
2	2	3	2	10.6	2	3	2
2	4	2	1	72.3	3	2	2
2	3	2	1	71.33	2	3	2
2	1	2	2	1.33	3	4	2
2	2	3	1	70.57	3	4	1
2	2	2	1	4	3	4	1
2	3	2	2	6	2	3	1
2	3	2	2	12.3	3	2	1
1	3	3	2	17.3	2	1	2
2	3	1	2	11.43	3	4	1
2	1	3	2	3.3	2	2	3
2	3	1	2	10.6	3	3	2
2	2	2	2	15.77	2	2	1
2	3	2	1	2.5	3	4	3
1	2	2	2	11.83	2	3	1
2	1	3	1	35.5	3	2	2
1	0	2	2	3.33	3	4	3
2	3	3	2	1.6	2	4	3
1	2	2	2	19	2	4	2
2	2	3	2	7.87	3	4	1
1	3	3	2	19.2	1	3	2
2	2	2	2	4.17	3	2	2
1	1	1	2	21	3	4	1
2	2	1	2	7.47	2	2	1
2	3	2	2	11.9	2	2	1
1	3	2	2	6.3	3	4	1
2	1	2	2	40.17	3	2	1
2	4	2	2	18.73	3	3	2
2	4	2	2	3.87	2	2	1
2	4	3	1	50.27	3	2	1
2	0	2	2	50.6	2	3	1
1	0	3	2	25.17	2	1	1
1	2	2	2	24.7	3	4	2
2	4	3	2	7.47	3	3	1
2	3	3	2	17.93	3	4	2
2	4	3	1	47.57	2	3	2
2	3	1	2	11.9	3	2	2
1	1	1	2	37.8	3	3	2
2	3	2	2	8.37	3	3	2
1	1	1	1	46.1	3	3	2
2	3	3	2	20.17	2	3	1
2	1	2	1	45.5	3	1	1
2	4	2	2	15.5	3	3	2
2	2	2	2	27.17	2	3	2
2	3	2	1	45.37	2	1	1
1	2	3	2	15.4	3	4	2

2	4	3	2	2.8	3	1	1
1	3	2	2	6.43	2	3	2
2	4	3	2	22.53	1	3	2
1	4	3	2	27.83	3	4	2
1	4	3	1	34.9	3	1	1
1	2	3	1	34.7	2	1	1
2	3	2	1	33.77	3	4	2
2	2	3	2	8.5	3	4	3
2	1	2	2	3.17	3	4	2
2	4	3	1	10.3	2	2	2
2	2	2	2	7.1	3	4	2
2	2	2	1	30.3	3	2	2
1	1	3	1	29.9	2	2	2
2	2	3	1	29.4	3	2	2
2	3	3	2	20.2	3	4	2
2	2	2	2	5.23	1	3	2
1	4	3	1	24.47	2	3	1
2	3	2	1	23.3	3	3	2
1	2	3	1	102.5	2	1	2
2	4	3	1	98.1	2	1	2
2	2	3	2	11.6	3	1	1
2	4	3	1	94.4	1	1	1
2	4	2	1	87.9	1	1	1
2	4	2	2	13.8	2	1	1
1	2	3	2	76.67	1	0	1
2	4	3	1	75.4	2	0	1
2	4	3	2	12.5	2	1	3
2	3	1	2	4.4	3	1	2
2	2	3	2	72.3	3	0	2
1	3	3	2	61.63	1	1	2
2	4	3	2	71.33	2	0	2
2	4	3	2	38.93	2	1	1
2	2	3	2	68.97	2	0	2
2	2	2	2	13.37	3	2	3
2	3	2	1	60.3	1	1	1
1	2	2	1	63.47	3	1	1
2	2	2	2	13.93	3	1	1
2	4	3	1	53.03	1	0	1
2	4	3	1	51.1	3	1	1
2	2	3	2	6.1	3	2	2
2	4	2	1	47.77	2	0	1
1	0	3	1	46.9	3	1	1
1	1	3	1	43.83	3	0	1
2	3	3	2	22.17	3	1	2
2	2	2	1	39.37	2	0	1
1	2	3	1	38.27	3	0	1
1	1	2	1	25.9	3	0	2
1	2	3	2	14.67	2	0	1
2	3	2	2	13.3	2	1	3
1	3	3	1	95.43	3	0	1
2	1	2	1	94.17	2	1	1
2	1	2	1	79.87	3	0	1
2	2	3	1	72.4	3	0	1
2	4	3	1	67.83	2	0	1

2	3	3	1	58.5	2	0	1
1	3	2	1	56.7	2	0	1
1	3	2	1	56.2	2	0	1
2	4	3	1	49.17	2	0	1
1	3	2	1	48.23	2	0	1
2	2	3	1	45.73	3	0	1
2	3	3	1	42.87	2	0	1
2	1	3	1	0.5	3	0	1
2	4	3	1	25.67	1	1	2
2	3	3	1	24.77	1	0	2
1	3	3	1	44.23	3	0	1
2	3	2					
1	1	3					
2	3	3					
2	3	2					
1	1	2					
2	4	3					
1	4	3					
2	2	3					
2	2	2					
2	3	2					
2	4	3					
2	3	2					
2	3	3					
2	0	2					
2	3	1					
2	3	3					
2	2	2					
1	2	2					
2	4	3					
2	1	3					
2	3	2					
1	2	2					
2	3	3					
1	2	3					
2	3	1					
2	3	3					
1	2	3					
2	4	3					
1	2	3					
1	1	3					
1	1	2					
2	1	3					
1	2	1					
1	4	3					
1	0	2					
2	4	3					
2	4	3					
2	3	3					
1	2	2					
1	0	3					
2	4	3					
2	2	3					
1	2	3					

1	2	2
1	2	3
2	4	3
2	3	1
1	3	2
1	2	3
1	4	2
1	3	3
1	1	2
2	0	2
1	1	2
2	1	3
1	1	1
2	0	2
2	2	1
2	1	1
1	1	1
1	1	3
2	1	2
1	1	2
1	1	1
2	2	3
2	0	3
2	0	1
1	0	2
1	1	2
1	0	2
2	0	3
2	1	2
2	1	3
1	1	2
2	0	3
2	1	1
2	0	2
2	0	3
2	0	2
1	0	3
1	2	2
2	1	2
2	0	2
2	0	3
2	2	3
2	0	2
2	2	3
1	1	3
1	0	3
1	1	1
2	0	2
1	0	3
1	0	3
1	1	3
1	0	2
1	0	3
1	0	3

1	0	3
2	0	2
1	1	2
2	1	3
1	1	2
1	0	2
1	0	1
1	2	2
2	0	3
1	0	3
1	1	3
1	0	2
2	1	3
1	1	2
1	1	3
1	2	2
2	2	3
2	0	2
1	0	1
1	0	2
2	0	2
1	1	2
1	1	3
1	1	3
1	1	2
1	1	3
1	0	3
1	0	2
2	0	2
1	0	3
1	0	3
2	0	2
1	0	3
1	1	3
1	0	3
2	1	2
2	1	3
1	0	2
2	0	3
2	2	3
2	1	1
1	0	3
1	0	2
1	0	2
1	0	3
2	2	3
2	2	3
1	1	2
1	0	2
1	1	2
2	0	3
1	0	3
2	0	3
1	1	3

2	0	2
2	1	2
1	0	2
1	0	3
1	1	2
1	0	2
1	1	2
1	0	2
1	0	3
1	0	2
1	1	2
1	0	2
1	0	3
1	0	3
1	0	3
1	0	2
1	0	2
1	0	2
1	0	2
1	1	2
1	0	3
1	0	2
2	0	2
1	0	3
1	0	2
1	1	3
1	0	2
1	0	3
1	0	2
1	0	3
1	0	3
1	0	2
1	0	2
1	0	2
1	0	2
1	0	2
1	0	3
1	0	2
1	1	3
1	0	3
1	0	2
1	0	3
1	0	2
1	0	3
1	0	2
1	0	3
1	0	3
1	0	2
1	0	2
2	0	2
1	0	3
1	0	2
1	0	1
1	1	1

1	0	3
1	0	3
1	0	1
2	1	3
2	0	2
1	0	2
1	0	2
1	0	2
1	1	3
1	0	1
1	0	2
1	0	2
1	0	2
1	0	3
1	0	3
1	0	3
1	0	3
1	0	2
1	0	1
1	0	3

反应组生存时间	反应组状态死亡2	反应组影像学评分	反应组阳性淋巴结分组		分组	无反应组影像学评分	无反应组时间	状态
0.5	1	1	1		3	1	106.5	1
15.63333	2	3	1		3	1	88.73	1
49.73333	2	2	1		3	1	84.8	1
45.73333	1	2	1		3	1	65.6	1
39.5	1	2	0		3	2	51.9	1
22.96667	1	3	4		3	2	42.53	2
42.6	2	2	0		3	2	51.47	1
59.23333	1	2	0		3	1	37.57	2
11.13333	2	2	4		3	1	74	1
14.76667	2	3	4		3	2	81.33	1
9	2	3	3		3	2	13.77	2
47.5	2	2	0		3	1	68.1	1
88.1	2	2	1		3	1	4.17	2
14.5	2	3	3		3	1	24.1	2
85.73333	1	2	0		3	2	27.57	2
20	2	2	2		3	3	6.47	2
23.66667	2	1	0		3	1	7.8	2
44.63333	2	1	2		3	2	18.33	2
18.3	2	1	4		3	2	17.7	2
59.96667	1	1	2		3	3	13.5	2
23.86667	2	2	4		3	2	46	2
22.5	2	2	3		3	1	68.5	2
13.3	1	1	2		3	1	24	2
54.03333	1	2	0		3	1	18	2
50	1	2	1		3	1	8.6	2
38.63333	2	2	1		3	3	10.17	2
20.13333	2	3	2		3	2	21.8	2
40.5	2	2	2		3	1	16.27	2
8.833333	2	3	3		3	1	74.87	2
40.36667	2	2	2		3	1	54.67	2
39.06667	1	1	2		3	1	21.5	2
30.1	2	2	3		3	1	12	2
15.9	2	2	1		3	2	7.97	2
33.36667	1	2	0		3	3	6.97	2
30.5	1	2	0		3	2	8.8	2
30	2	2	2		3	1	90.53	1
91.1	2	1	0		3	1	6	2
75.53333	1	1	1		3	2	86.5	1
61.26667	1	1	1		3	2	52	2
18.03333	2	2	0		3	1	84.8	1
59.66667	1	1	0		3	1	1.47	2
50	1	1	0		3	2	5.37	2
49	1	2	1		3	1	4.2	2
48.76667	1	1	2		3	2	7.5	2
49.6	1	1	0		3	2	49.43	2
47.03333	1	1	1		3	1	25.63	1
20.33333	2	1	0		3	2	77.13	1
44.3	1	1	0		3	2	18.5	2
20	2	3	0		3	2	38.1	2

34.56667	1	2	0	3	2	43.33	2
33.13333	2	2	2	3	3	2.63	2
29.8	1	2	1	3	2	50.97	1
98.13333	1	2	0	3	2	41.93	2
50.9	1	1	0	3	2	48	1
88.56667	1	1	1	3	1	18.37	2
87.33333	1	1	0	3	3	16	2
85.83333	1	1	0	3	3	16.57	2
85.5	1	1	0	3	2	10.6	2
79.93333	1	1	0	3	2	72.3	1
34.5	1	1	0	3	2	71.33	1
60.3	1	1	0	3	2	1.33	2
59.03333	1	2	0	3	1	70.57	1
53.06667	1	1	0	3	1	4	1
50.53333	1	2	0	3	1	6	2
50.53333	1	2	0	3	1	12.3	2
50.26667	1	2	0	3	2	17.3	2
49.66667	1	2	0	3	1	11.43	2
47.8	1	2	0	3	3	3.3	2
47.13333	1	1	0	3	2	10.6	2
39.96667	1	1	0	3	1	15.77	2
39.8	1	2	0	3	3	2.5	1
29.93333	1	2	0	3	1	11.83	2
29.7	1	1	0	3	2	35.5	1
25.1	1	2	0	3	3	3.33	2
25.13333	1	2	0	3	3	1.6	2
24.46667	2	2	1	3	2	19	2
22.33333	2	3	0	3	1	7.87	2
23.33333	1	2	0	3	2	19.2	2
37.9	1	2	0	3	2	4.17	2
7.5	1	1	0	3	1	21	2
75.1	1	1	0	3	1	7.47	2
64.06667	1	1	0	3	1	11.9	2
54.36667	1	3	0	3	1	6.3	2
47.5	1	1	0	3	1	40.17	2
45.5	1	2	0	3	2	18.73	2
37.33333	1	2	0	3	1	3.87	2
32	1	2	0	3	1	50.27	1
26.93333	1	1	0	3	1	50.6	2
				3	1	25.17	2
				3	2	24.7	2
				3	1	7.47	2
				3	2	17.93	2
				3	2	47.57	1
				3	2	11.9	2
				3	2	37.8	2
				3	2	8.37	2
				3	2	46.1	1
				3	1	20.17	2
				3	1	45.5	1
				3	2	15.5	2
				3	2	27.17	2
				3	1	45.37	1
				3	2	15.4	2

3	1	2.8	2
3	2	6.43	2
3	2	22.53	2
3	2	27.83	2
3	1	34.9	1
3	1	34.7	1
3	2	33.77	1
3	3	8.5	2
3	2	3.17	2
3	2	10.3	1
3	2	7.1	2
3	2	30.3	1
3	2	29.9	1
3	2	29.4	1
3	2	20.2	2
3	2	5.23	2
3	1	24.47	1
3	2	23.3	1
3	2	102.5	1
3	2	98.1	1
3	1	11.6	2
3	1	94.4	1
3	1	87.9	1
3	1	13.8	2
3	1	76.67	2
3	1	75.4	1
3	3	12.5	2
3	2	4.4	2
3	2	72.3	2
3	2	61.63	2
3	2	71.33	2
3	1	38.93	2
3	2	68.97	2
3	3	13.37	2
3	1	60.3	1
3	1	63.47	1
3	1	13.93	2
3	1	53.03	1
3	1	51.1	1
3	2	6.1	2
3	1	47.77	1
3	1	46.9	1
3	1	43.83	1
3	2	22.17	2
3	1	39.37	1
3	1	38.27	1
3	2	25.9	1
3	1	14.67	2
3	3	13.3	2
3	1	95.43	1
3	1	94.17	1
3	1	79.87	1
3	1	72.4	1
3	1	67.83	1

3	1	58.5	1
3	1	56.7	1
3	1	56.2	1
3	1	49.17	1
3	1	48.23	1
3	1	45.73	1
3	1	42.87	1
3	1	0.5	1
3	2	25.67	1
3	2	24.77	1
3	1	44.23	1
1	1	0.5	1
1	3	15.63333	2
1	2	49.73333	2
1	2	45.73333	1
1	2	39.5	1
1	3	22.96667	1
1	2	42.6	2
1	2	59.23333	1
1	2	11.13333	2
1	3	14.76667	2
1	3	9	2
1	2	47.5	2
1	2	88.1	2
1	3	14.5	2
1	2	85.73333	1
1	2	20	2
1	1	23.66667	2
1	1	44.63333	2
1	1	18.3	2
1	1	59.96667	1
1	2	23.86667	2
1	2	22.5	2
1	1	13.3	1
1	2	54.03333	1
1	2	50	1
1	2	38.63333	2
1	3	20.13333	2
1	2	40.5	2
1	3	8.833333	2
1	2	40.36667	2
1	1	39.06667	1
1	2	30.1	2
1	2	15.9	2
1	2	33.36667	1
1	2	30.5	1
1	2	30	2
1	1	91.1	2
1	1	75.53333	1
1	1	61.26667	1
1	2	18.03333	2
1	1	59.66667	1
1	1	50	1
1	2	49	1

1	1	48.76667	1
1	1	49.6	1
1	1	47.03333	1
1	1	20.33333	2
1	1	44.3	1
1	3	20	2
1	2	34.56667	1
1	2	33.13333	2
1	2	29.8	1
1	2	98.13333	1
1	1	50.9	1
1	1	88.56667	1
1	1	87.33333	1
1	1	85.83333	1
1	1	85.5	1
1	1	79.93333	1
1	1	34.5	1
1	1	60.3	1
1	2	59.03333	1
1	1	53.06667	1
1	2	50.53333	1
1	2	50.53333	1
1	2	50.26667	1
1	2	49.66667	1
1	2	47.8	1
1	1	47.13333	1
1	1	39.96667	1
1	2	39.8	1
1	2	29.93333	1
1	1	29.7	1
1	2	25.1	1
1	2	25.13333	1
1	2	24.46667	2
1	3	22.33333	2
1	2	23.33333	1
1	2	37.9	1
1	1	7.5	1
1	1	75.1	1
1	1	64.06667	1
1	3	54.36667	1
1	1	47.5	1
1	2	45.5	1
1	2	37.33333	1
1	2	32	1
1	1	26.93333	1
2	1	52.06667	1
2	2	51.9	2
2	1	52.03333	1
2	1	51.96667	1
2	2	51.2	1
2	1	51.4	1
2	1	92.43333	1
2	1	35.7	2
2	3	43.5	1

2	1	35.9	2
2	1	5.766667	2
2	1	15.63333	2
2	3	16.33333	2
2	2	34.8	2
2	2	11.5	2
2	3	8	2
2	1	96.1	1
2	2	8.433333	2
2	3	2.6	2
2	3	5.533333	2
2	1	30.46667	2
2	2	16.2	2
2	1	85.5	1
2	3	14.9	2
2	1	82.83333	1
2	1	82.13333	1
2	2	50.93333	2
2	1	38.26667	2
2	2	70.1	1
2	1	67.4	2
2	1	65.03333	1
2	2	14.5	2
2	1	23.9	2
2	1	58.76667	1
2	2	15.56667	2
2	2	6.566667	2
2	1	20.46667	2
2	1	54.06667	1
2	1	49.83333	1
2	2	49.43333	1
2	2	32.2	2
2	1	48.3	1
2	1	48.5	1
2	1	22.96667	2
2	1	11.53333	2
2	2	10.56667	2
2	2	49.5	1
2	1	36.16667	2
2	3	13.83333	2
2	3	10.53333	2
2	1	27.66667	2
2	2	30.3	2
2	1	20.4	2
2	2	40.23333	1
2	2	37.86667	1
2	2	33.13333	2
2	1	37.5	1
2	2	37.2	1
2	2	34.86667	1
2	3	13.33333	2
2	1	23.8	1
2	1	23.56667	1
2	2	23.5	2

2	2	16.4	2
2	1	67.33333	2
2	1	87.93333	1
2	1	83.13333	1
2	1	80.63333	1
2	1	66.8	2
2	1	1.433333	2
2	1	76.6	1
2	1	42.26667	2
2	2	23.8	1
2	1	29.4	2
2	1	25.2	2
2	2	69.2	1
2	1	69.16667	1
2	2	32.63333	2
2	3	19.13333	2
2	2	24.2	2
2	1	60.76667	1
2	1	59.7	1
2	1	58.23333	1
2	1	57.4	1
2	2	57.4	1
2	2	57.96667	1
2	1	24.26667	2
2	2	54.2	1
2	2	53.83333	1
2	1	54.6	1
2	2	54	1
2	1	15.93333	2
2	1	51.06667	1
2	2	35.66667	2
2	2	49.1	1
2	2	31.6	2
2	3	7.033333	2
2	1	47.36667	1
2	2	46.23333	1
2	1	45.86667	1
2	1	45.9	1
2	2	44.7	1
2	1	44.73333	1
2	1	27	2
2	2	41.13333	1
2	1	41.03333	1
2	3	12.96667	2
2	2	31.36667	2
2	2	5.566667	2
2	2	37.73333	1
2	2	34.33333	1
2	2	30.8	2
2	1	30.8	1
2	2	30.6	1
2	2	26.03333	2
2	2	22.23333	2
2	2	25.36667	1

2	1	85.16667	1
2	1	72.73333	1
2	1	71.86667	1
2	2	59.5	1
2	1	59.03333	2
2	1	57.53333	1
2	2	56.46667	1
2	2	52.53333	1
2	1	49.63333	1
2	1	47.06667	1
2	1	44.8	1
2	2	43.56667	1
2	1	39.46667	1
2	2	12	1
2	2	33.9	1
2	3	10.73333	2
2	1	29.43333	1
2	2	23.43333	1
2	1	23.3	1
2	1	22.9	1
